# Intracellular common gardens reveal niche differentiation in transposable element community during bacterial adaptive evolution

**DOI:** 10.1038/s41396-022-01344-2

**Published:** 2022-11-24

**Authors:** Hui Guo, Wen-Tao Shi, Biliang Zhang, Yan-Hui Xu, Jian Jiao, Chang-Fu Tian

**Affiliations:** 1 State Key Laboratory of Agrobiotechnology, and College of Biological Sciences, China Agricultural University, Beijing, China; 2 MOA Key Laboratory of Soil Microbiology, and Rhizobium Research Center, China Agricultural University, Beijing, China

**Keywords:** Molecular ecology, Molecular evolution, Mutation, Community ecology

## Abstract

The distribution and abundance of transposable elements across the tree of life have significantly shaped the evolution of cellular organisms, but the underlying mechanisms shaping these ecological patterns remain elusive. Here we establish a “common garden” approach to study causal ecological interactions between a xenogeneic conditional lethal *sacB* gene and the community of transposable insertion sequences (ISs) in a multipartite prokaryote genome. Xenogeneic *sacB* of low, medium, or high GC content was individually inserted into three replicons of a model bacterium *Sinorhizobium fredii*, and exhibited replicon- and GC-dependent variation in genetic stability. This variation was largely attributable to multidimensional niche differentiation for IS community members. The transposition efficiency of major active ISs depended on the nucleoid-associated xenogeneic silencer MucR. Experimentally eliminating insertion activity of specific ISs by deleting MucR strongly demonstrated a dominant role of niche differentiation among ISs. This intracellular common garden approach in the experimental evolution context allows not only for evaluating genetic stability of natural and synthetic xenogeneic genes of different sequence signatures in host cells but also for tracking and testing causal relationships in unifying ecological principles in genome ecology.

## Introduction

Transposable elements (TEs), discovered in 1940s, have been intensively studied regarding their molecular properties for the past 70 years [1–3]. This is largely due to their significant contribution to genome size variation and adaptive evolution of cellular organisms across the tree of life [4, 5], and their ever-increasing value for the development of forward genetics and reverse genome editing tools [6–9]. However, the processes shaping the distribution and abundance of TEs, between genomes and within a genome, remain elusive.

Analogies have been drawn between TEs within host genomes and organisms within an environment, giving rise to the concept of genome ecology, where TEs are analogous to organisms [10–14]. Therefore, adapted from the conventional definition of ecology [15], genome ecology of TEs is the study of the distribution and abundance of TEs and the interactions that determine distribution and abundance. Interspecific and intraspecific host variations in cellular parameters define ecological niches for TEs, and affect transposition and deletion rates, which are analogous to birth and death rates, respectively [12, 16]. TE distribution and abundance between and within host species have been described and interpreted in terms of genomic niche (such as target sequence features, TE positions and copy numbers) [13, 17], demography [18–20], interspecific competition [21, 22], predator-prey dynamics [22, 23], ecological neutral theory [24], and genome ecosystem [25]. These pioneering efforts have revealed intriguing in situ correlations in the context of TE ecology. By contrast, a “common garden” approach, widely used in testing causal ecological relationships underlying geographic variation of organism distribution and abundance [15], has not been effectively integrated into the toolbox of genome ecology. Common garden experiments involve the comparison of genetically distinct entities under identical conditions to disentangle the effects of genetic and environmental variation.

A core tenet of ecology is to understand resource requirements and tolerance of organisms in the *n*-dimensional hypervolume niche, which is a situation rather than a place [15]. We aimed to experimentally test the hypothesis that genomic niche differentiation affects the rate of insertion by different TEs in the context of intracellular ecology (Table 1). To this end, we focused on a model bacterium, *Sinorhizobium fredii* CCBAU25509 (hereafter SF2), that is a facultative microsymbiont of soybean plants, living saprophytically in soils and differentiating into nitrogen-fixing bacteroids inside root nodule cells of compatible soybean plants in the nitrogen-depleted soil [26–28]. SF2 harbors three replicons with contrasting GC content and patterns of TE distribution and abundance [26, 27], and can be considered as a well-bounded ecosystem with three habitats (Table 1). It was recently demonstrated that insertion mutations, mainly mediated by a pool of transposable insertion sequences (ISs), within a low GC% gene cluster encoding the type three secretion system (T3SS) and its effector protein NopP allowed fast evolution of SF2 into a compatible microsymbiont of certain commercial soybean cultivars [27]. However, working with numerous T3SS genes as baits to capture mobile ISs is time-consuming (two months), laborious, and expensive in screening insertion mutations mediated by ISs.

**Table 1:** Analogies between intracellular ecology of mobile elements and conventional ecology.

Conventional ecology	Intracellular ecology
Organisms	Mobile insertion sequences (ISs)
Well-bounded ecosystem	Bacterial cell (*S. fredii* CCBAU25509)
Different habitats	Different replicons (Ch, pB, pA)
Common gardens	IS insertion targets under selection (*sacB* in different replicons)
Niche dimensions	Intracellular variables (e.g. replicon GC%, *sacB* GC%, SacB enzyme activity, available insertion sites in *sacB*, and available ISs in each replicon)

In this work, a xenogeneic conditional lethal gene *sacB* was individually inserted into each replicon (habitat) to trap mobile ISs during adaptive evolution. Three *sacB* versions of low, medium, or high GC% in synonymous codons were used, resulting nine independent replicon-*sacB* pairs which were considered as nine common gardens for intracellular IS community members (Table 1). Successful insertion events in *sacB* of these common gardens led to a flourishing cellular ecosystem under a selecting condition, which allowed further identification of captured ISs by routine sequencing of PCR products. Niche delineation analysis was then used to uncover intracellular variables shaping the observed variation of IS insertion efficiency across common gardens. The major active ISs preferred xenogeneic *sacB* of low and medium GC% in a low GC% replicon, and this observed niche differentiation was then uncovered as a process depending on a xenogeneic silencer MucR conserved in *Alphaproteobacteria* [29, 30]. The significance of this common garden approach in uncovering causal relationships in the context of genome ecology was demonstrated, and discussed in the context of evolutionary and synthetic biology.

## Materials and methods

### Bacterial strains, primers, and growth conditions

Bacterial strains, plasmids, and primers used in this study are shown in Supplementary Table S1. *Escherichia coli* strains carrying plasmids used in conjugation experiments were grown at 37 °C in LB medium. *S. fredii* CCBAU25509 (SF2) and its derivatives were grown at 28 °C in TY medium (5 g tryptone, 3 g yeast extract, 0.6 g CaCl_2_ per liter). To screen and purify conjugants or obtain pure cultures of bacteria, antibiotics were supplemented as required at the following concentrations (μg/mL): for *E. coli*, gentamicin (Gen), 30; and kanamycin (Km), 100; for *Sinorhizobium* strains, trimethoprim (Tmp), 10; nalidixic acid (Na), 30; and kanamycin (Km), 100. To screen *sacB* mutants from SF2 derivatives, firstly SF2 tolerance of 8%-30% sucrose in the TY medium was measured by the growth curve using Bioscreen C (Oy Growth Curves Ab Ltd, Raisio, Finland), and then the TY medium containing 10% sucrose was chosen as the selection medium.

### Construction of *S. fredii* derivatives harboring xenogeneic PsacB-*sacB*

**Fig. 1:**
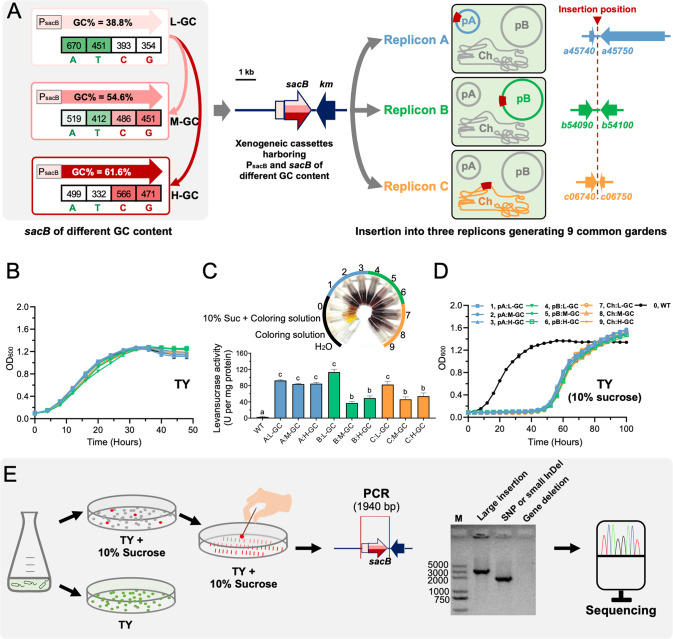
Screening mutations in xenogeneic *sacB* of different GC content. **A** The xenogeneic cassettes harboring *sacB* of L-GC, M-GC, or H-GC were individually inserted into the symbiosis plasmid (pA; GC% = 59%), chromid (pB; GC% = 62%), or chromosome (Ch; GC% = 62.6%) of *Sinorhizobium fredii* CCBAU25509. Gene IDs surrounding each insertion position are shown. GC% of the three *sacB* versions were 38.8% (L-GC, the wild-type version from *Bacillus subtilis* subsp. subtilis str. 168), 54.6% (M-GC, synthesized), and 61.6% (H-GC, synthesized). The wild-type PsacB (GC% = 36.1%, 446 bp) of *B. subtilis* 168 was cloned together with each of the three versions of *sacB*. The number of A, T, C, or G in the 1422 bp *sacB* gene is indicated. **B** Growth curves in TY medium. **C** Levansucrase enzyme activity assay of crude proteins collected at OD_600_ = 1.2 in TY medium. Different letters indicate significant difference (Average ± SEM; ANOVA followed by Duncan’s test, alpha = 0.05). **D** Growth curves in TY medium supplemented with 10% sucrose. **E** Schematic view of culturing, mutant screening, and mutation identification in this work. *sacB*, levansucrase gene; *km*, kanamycin resistance gene.

The multipartite genome of SF2 consists of a chromosome (Ch, GC% = 62.6%), a chromid (pB, GC% = 62%) [31], and a symbiosis plasmid (pA, GC% = 59%) [26]. Within each replicon, an insertion position, with GC% of its 10 kb flanking region being the same as the replicon average, was chosen for subsequent experiments (Fig. 1A). The suicide plasmid pJQ200SK carries the wild-type *sacB* gene (characterized by its low GC content of 38.8%; 1422 bp) and its promoter region PsacB (GC% = 36.1%, 446 bp) from *Bacillus subtilis* subsp. subtilis str. 168 [32]. A Km-resistant cassette from pBBR1MCS-2 [33] was amplified and assembled with a linearized pJQ200SK lacking the Gm-resistant cassette using a seamless cloning kit (Taihe Biotechnology, Beijing, China) as described previously [34]. This generated pJQ-L carrying the wild-type low GC% *sacB* (38.8%; 1422 bp; L-GC). The *sacB* gene with medium (54.6%; M-GC) or high GC (61.6%; H-GC) content in its synonymous codons was synthesized (Fig. S1), and used to replace the wild-type low GC% *sacB* gene of pJQ-L to generate pJQ-M and pJQ-H. This was also performed using the seamless cloning method as described above with the linearized pJQ-L lacking the wild-type *sacB*. Three genomic segments of SF2 (pA:330682-331687, pB:702541-703493, Ch:674057-675207) were individually cloned into each of pJQ-L, pJQ-M, and pJQ-H at the SmaI site using the seamless cloning method, which allowed subsequent integration of xenogeneic cassettes into three replicons. This generated nine plasmids (pJQ-L_pA, pJQ-L_pB, pJQ-L_Ch; pJQ-M_pA, pJQ-M_pB, pJQ-M_Ch; pJQ-H_pA, pJQ-H_pB, pJQ-H_Ch), which were transformed into *E. coli* DH5α and verified by Sanger sequencing before conjugation into rhizobia via triparental mating with helper plasmid pRK2013 [35]. This generated nine SF2 derivatives individually carrying a xenogeneic cassette in a replicon (Fig. 1A). The correct insertion of the xenogeneic cassette was checked by PCR.

The xenogeneic silencer MucR prefers low GC% DNA targets [29, 30], and its potential role in niche differentiation for IS community members was tested. SF2 has two *mucR* copies, and the in-frame deletion mutant Δ*mucR1R2* was constructed by using an allelic exchange strategy: upstream and downstream ~500 bp flanking regions of *mucR1* or *mucR2* were amplified and assembled with the linearized allelic exchange vector pJQ200SK. The pJQ200SK derivative used to delete *mucR1* was linearized and then cloned seamlessly with the sequence coding MucR1 and C-terminal fused FLAG-tag. The resultant plasmid was conjugated into SF2 to generate SF2MucR1FLAG. The xenogeneic cassettes carrying plasmids (pJQ-L_pA, pJQ-M_pA, pJQ-H_pA) were then inserted into the same position of pA in Δ*mucR1R2* and SF2MucR1FLAG, and verified by PCR.

### Mutant screening and calculation of mutation frequency

To screen *sacB* mutants from SF2 derivatives, single colonies of *S. fredii* derivatives were inoculated and grown to an OD_600_ = 0.2, 0.6, 1.2, and 2.0, and dilutions were applied to plates with and without 10% sucrose respectively. The number of colonies on the 10% sucrose TY plates was recorded as “A” at the dilution of 10^−a^, and the number of colonies on the sucrose-free TY plates was recorded as “B” at the dilution of 10^−b^. The total mutation frequency was then calculated by (A·10^-a^)/(B·10^-b^). Independent colonies on the 10% sucrose TY plates were further purified on the same medium plates, and the full length of PsacB-*sacB* fragment was amplified by colony PCR. Gene loss, SNPs or short InDels, or large insertion mutations were identified by electrophoresis analysis of PCR products. Representative clones with large insertion mutations were selected for Sanger sequencing. Three independent experiments were performed for all test strains.

### Enzyme activity assay for levansucrase

To evaluate the function of xenogeneic *sacB* in SF2 derivatives, sucrose was dissolved in the buffer solution (0.1 M CH_3_COONa, pH 5.5), and the total protein extract of bacteria was added (calibrated to the same concentration) to make the final concentration of sucrose 1%, and the reaction system was incubated at 28°C for 12 h. After adding the color development solution (3,5-dinitrosalicylic acid 6.3 g, sodium hydroxide 21.0 g, potassium sodium tartrate 182.0 g, phenol 5.0 g, sodium metabisulfite 5.0 g in 1000 mL water; BOXBIO, Beijing, China), the enzyme was inactivated at 95 °C for 5 min, and the absorbance value at 540 nm was measured to calculate the glucose content. Determination of the release of glucose and fructose from sucrose allowed calculation of the total activity of the levansucrase. One unit (U) of enzyme is defined as the amount of enzyme required for producing 1 µmol glucose per min in reaction buffer. The specific activity of levansucrase hydrolysis activity is the activity units per mg of protein (U/mg).

### 5′RACE

To determine the transcription start site of the *sacB* gene, a 5′RACE experiment was performed with the 5′RACE kit (Sangon, Beijing, China) for Rapid Amplification of cDNA Ends using three gene-specific primers (Table S1) that anneal to the known region and an adapter primer that targets the 5′ end. Products generated by 5′RACE were subcloned into the TOPO-TA vector and individual colonies were sequenced.

### RNA extraction and RT-qPCR

To determine transcriptional levels of the major active ISs in SF2 and its Δ*mucR1R2* mutant, strains were grown in 50 mL TY liquid medium to an OD_600_ of 1.2. A bacterial total RNA Kit (Zomanbio, Beijing, China) was used for total RNA extraction. cDNA was synthesized using FastKing-RT SuperMix (TIANGEN, Beijing, China). qPCR was performed by using QuantStudio 6 Flex and 2× RealStar Green Mixture (Genstar, Beijing, China). The primer pairs used are listed in Table S1. The 16S rRNA gene was used as an internal reference to normalize the expression level. Three independent biological replicates were performed.

### ChIP-qPCR

To test the potential recruitment of MucR in the xenogeneic PsacB-*sacB* region, three SF2 derivative strains harboring *sacB* of different GC% in the pA replicon and MucR1-FLAG (Table S1; MucR1-FLAG: L-GC, MucR1-FLAG: M-GC, MucR1-FLAG: H-GC) were cultured until the OD_600_ had reached 1.2. Formaldehyde was added into the TY medium to a final concentration of 1%, which was then incubated at 28 °C for 15 min. To stop crosslinking, glycine was added to a final concentration of 0.1 M. The cross-linked samples were harvested (5000 × *g*, 5 min, 4 °C) and washed twice with cold phosphate-buffered saline (PBS). After the pellets were ground into fine powder in liquid nitrogen, the samples were resuspended in buffer containing 1% SDS and 1 mM phenylmethanesulfonyl fluoride, and lysed by sonication using a sonicator (Q800R3, QSonica). Chromatin immunoprecipitation (ChIP) was performed using the ChIP assay kit (Beyotime, Shanghai, China) according to the manufacturer’s recommendations. The supernatant was collected and chromatin was immunoprecipitated with Anti-FLAG M2 antibody (Sigma). Input control and DNA obtained from the immunoprecipitation were amplified by PCR using primers listed in Table S1. The recruitment level of FLAG-tagged MucR1 in multiple regions within the PsacB-*sacB* fragment inserted by ISs at high frequency was detected by ChIP-qPCR.

### Crosslinking and western blotting assay

To test the ability of MucR1 to form homodimer in SF2 derivatives carrying *sacB* in pA, rhizobial cells (SF2MucR1FLAG, MucR1-FLAG: L-GC, MucR1-FLAG: M-GC, and MucR1-FLAG: H-GC) were cultured in 50 mL TY medium to an OD_600_ of 1.2. Formaldehyde was added at a final concentration of 1% in the culture which was then shaken at 28 °C, 100 rpm for 15 min to allow crosslinking. The crosslinking reaction was terminated by adding a final concentration of 100 mM glycine (28 °C, 100 rpm, 5 min). 1 mL of the above solution was centrifuged (5000 × *g*, 4 °C, 1 min), resuspended in 50 µL SDS loading buffer to a uniform cell density, and then boiled for 10 minutes for lysis. Next, lysates were separated on 12% SDS-PAGE and transferred to a nitrocellulose membrane. For immunodetection of individual proteins, the method described previously was used [30]. Briefly, mouse monoclonal Anti-FLAG M2 antibody (Sigma), HRP (horseradish peroxidase) conjugated goat Anti-mouse IgG (Abcam), and eECL Western blot kit (CWBIO, Beijing, China) were used, and chemiluminescence signals were visualized using Fusion FX6 (Vilber) and Evolution-Capt Edge software.

### Protein purification

To purify MucR1 protein, *E. coli* BL21(DE3) carrying His_6_-SUMO-tagged MucR1 in the pET30a [29] was cultured in 500 mL LB medium until OD_600_ reached 0.8. The procedure described previously was used [30]. IPTG was then added to the culture to a final concentration of 0.6 mM and switched to 18 °C at 150 rpm for 12 h. Cells were harvested by centrifugation (5000 × *g*, 5 min, 4 °C) and resuspended in 30 mL of lysis buffer (25 mM Tris, pH 8.0, 250 mM NaCl, 10 mM imidazole) supplemented with 0.1 mg/mL DNase I, 0.4 mg/mL of lysozyme, and protease inhibitor mixture (Roche). After 30 min incubation and 120 sonication cycles (300 W, 10 s on, 10 s off), lysates were removed by centrifugation (18,000 × *g*, 4 °C, 30 min) and filtration through a 0.22 μm membrane. The supernatant was loaded onto Ni-Agarose Resin (CWBIO, Beijing, China) pre-washed using lysis buffer, washed 3 times with wash buffer (lysis buffer containing 20 mM imidazole), and then eluted by lysis buffer containing imidazole gradient (100, 200, 300 mM imidazole). The purified proteins were finally concentrated by ultrafiltration and redissolved in storage buffer (25 mM Tris, pH 8.0, 250 mM NaCl, 10% glycerol) prior to use or storage at −80 °C.

### DNA bridging assay

To determine if MucR1 can form DNA-MucR1-DNA complex with various regions of xenogeneic PsacB-*sacB* fragment, a DNA bridging assay described earlier [30, 36] was performed with modifications. DNA probes were prepared by annealing of synthesized complementary strands (PsacB −90~−24) or by PCR amplification (PsacB −90~+3, *sacB* +710~+802, *sacB* +908~+1007) using 5′-biotin-labeled or 5′-Cy5 primers (Table S1). In each bridging assay, 100 μL of hydrophilic streptavidin magnetic beads (NEB) were washed twice with 500 μL of PBS and then resuspended in 500 μL of coupling buffer (20 mM Tris-HCl, pH 7.4, 1 mM EDTA, 500 mM NaCl). Then, the suspension was supplied with 10 pmol of biotin-labeled DNA and incubated with the beads for 30 min at room temperature with gentle rotation. The resulting beads were washed twice with 500 μL of incubation buffer (20 mM Tris, pH 7.4, 150 mM NaCl, 1 mM dithiothreitol, 5% glycerol (vol/vol), 0.05% Tween 20) and resuspended after the addition of 10 pmol Cy5-labeled DNA and 10 μL HRV 3C protease to a final volume of 500 μL. The HRV 3C protease was used herein to remove SUMO. A twofold serial dilution of the protein sample was added to each 50 μL aliquot of bead suspension, and supplemented with incubation buffer to 60 μL final volume. After 30 minutes of incubation with gentle rotation at room temperature, the mixture was placed on a magnetic stand for 5 minutes. The supernatant was collected and labeled as Sample A. The beads were mixed with 60 μL of elution buffer (incubation buffer with 0.1% SDS and 20 μg/mL biotin) and incubated in a boiling water bath for 10 min. The eluted samples were labeled as Sample B. Cy5 fluorescence signals of Sample A and B were detected by a Microscale Thermophoresis Monolith NT.115 system (NanoTemper). The Cy5 fluorescence signal of the Sample A from the treatment without MucR1 was defined as 100% input signal.

### Statistical analyses

Analysis of variance (ANOVA) followed by Duncan’s test, Student’s t-test, and Fisher’s exact test were performed using GraphPad Prism 8. The closest homolog of individual active ISs and their family identification were determined using ISfinder [37]. Target sequence logos of ISs were generated by multiple sequence alignments of insertion sites within xenogeneic PsacB-*sacB* or genomic background using the program WebLogo [38].

Although the fundamental niche, not constrained by biological interactions, cannot be determined by observation [15], the realized niche, representing a proportion of the fundamental niche where organisms actually live under abiotic and biotic interactions, can be estimated by correlative approaches [15, 39]. In order to address the influence of intracellular variables on biased IS insertions into nine common gardens, the within outlying mean index analysis developed for niche differentiation analysis was carried out using the R package “subniche” [40, 41]. The intracellular environmental gradients were determined by Principal Component Analysis (PCA) based on variables as follows: GC% of different *sacB* versions, replicon GC%, the number of each IS in the corresponding replicon where *sacB* is inserted, available insertion sites of ISs in different *sacB* versions, and levansucrase activity of strains carrying different *sacB* versions. Within this multidimensional Euclidean space (environmental space), mean positions in realized (sub)niches and parameters of each IS were obtained for the whole data set (realized niches in environmental space defined by nine common gardens) or various subsets (realized subniches in sub-environmental spaces identified by the hierarchical clustering analysis with the *ward.D* method based on the Euclidean distance matrix) [41]. Two and three subsets rather than four and more subsets were statistically analyzable. By comparing to the overall average habitat conditions (*G*) or the average subset habitat conditions (*G*_*K*_) of the spatial domain, ISs selecting for a less common habitat were indicated by their significantly higher niche marginality values compared to the simulated values, based on a Monte Carlo test with 1,000 permutations, under the hypothesis that each IS is indifferent to its intracellular environment [40].

## Results

### Screening *sacB* mutations from nine intracellular common gardens

Nine SF2 derivatives carrying different *sacB* versions (Fig. 1A and Table S1) showed indistinguishable growth curves in the TY rich medium (Fig. 1B) and expressed the enzymatic activity of levansucrase encoded by xenogeneic *sacB* (Fig. 1C; converting sucrose into fructose and glucose that can be detected by a color reaction). In the presence of 10% sucrose in the TY rich medium, no notable growth was observed for these nine derivatives harboring *sacB* until the wild-type SF2 entered into stationary phase (Fig. 1D). By contrast, a strong growth inhibition effect was only observed for SF2 when 30% sucrose was supplied (Fig. S2A). Therefore, xenogeneic *sacB* conferred a conditional deleterious effect for SF2 derivatives in the presence of 10% sucrose, and the observed later recovery of all nine derivatives under the same conditions (Fig. 1D) might be mediated by potential spontaneous mutations in *sacB* under the strong selection pressure. To test this hypothesis and determine frequency of potential mutation events, bacterial cultures at different growth stages in the liquid TY medium (OD_600_ = 0.2, 0.6, 1.2, or 2.0) were plated on the TY plate containing 10% sucrose to screen independent *sacB* mutants, and on the control TY plate to count the total number of cells under selection (Fig. 1E). The *sacB* mutation events were then identified as large insertions, SNPs or small InDels, and gene deletions based on PCR amplification using primers targeting the full length of PsacB and the coding region of *sacB* (Fig. 1E). PCR products of those mutants with large insertions in the xenogeneic PsacB-*sacB* region were subject to Sanger sequencing to identify putative TEs.

### Replicon- and GC-dependent genetic stability of xenogeneic *sacB*

**Fig. 2:**
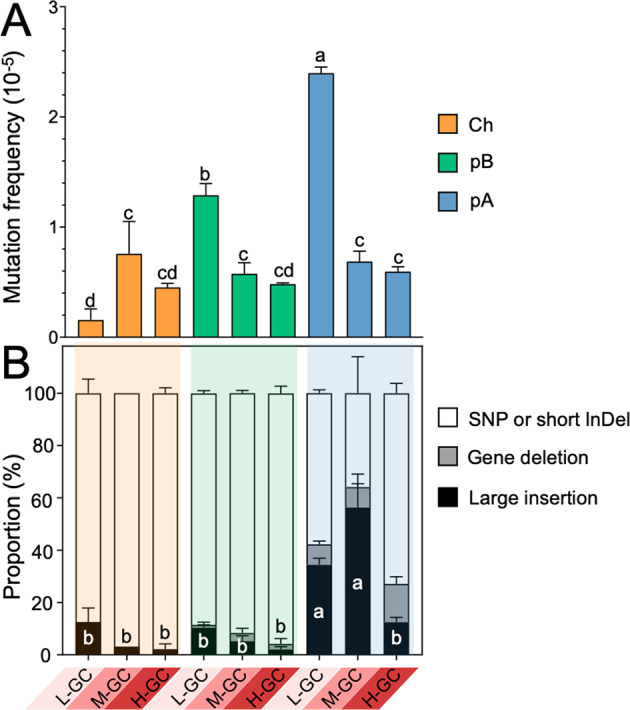
Replicon- and GC-dependent variation in mutation frequency and mutation mechanisms. **A** Total mutation frequency of *sacB* in stationary phase (OD_600_ = 1.2). **B** Proportions of mutation events mediated by different mutation mechanisms: large insertions, gene deletions, SNPs, and/or small InDels. Three independent experiments were performed. Different letters indicate significant difference (Average ± SEM; ANOVA followed by Duncan’s test, alpha = 0.05) between mutation frequency values (**A**) or between proportion values for large insertion events (**B**). Similar results were obtained from samples collected at OD_600_ = 0.2, 0.6, and 2.0 (Fig. S2B, C).

Based on three independent experiments, it was found that frequency of mutation of *sacB*, and the mutation mechanisms involved, were both GC- and replicon-dependent (Fig. 2A, B and Fig. S2B, C; ANOVA followed by Duncan’s test, alpha = 0.05). L-, M-, and H-GC versions in the chromosomal replicon (Ch) all exhibited a low mutation frequency throughout four test stages (frequency below 8 × 10^−6^ for cultures of OD_600_ = 0.2, 0.6, 1.2, or 2.0; Fig. 2A and Fig. S2B). In the pB replicon, the L-GC *sacB* version had a mutation frequency twice those of the M-GC and H-GC versions, which were at similar levels to those of the corresponding *sacB* versions in the Ch replicon. In the pA replicon, the L-GC *sacB* version had a mutation frequency more than twice those of the M-GC and H-GC versions, and the mutation frequency in the M-GC version was significantly higher than in the H-GC version for exponential phase cultures (OD_600_ = 0.2 and 0.6; Fig. S2B). As to the mutation mechanisms (Fig. 2B and Fig. S2C), in contrast to the *sacB* versions in Ch or pB, those in pA were more prone to mutation mediated by large insertions as well as gene deletions, with a particularly high proportion of large insertion mutations in L-GC and M-GC versions. In Ch or pB, the L-GC *sacB* version generally had a higher proportion of mutations mediated by large insertions than M-GC or H-GC, though not always statistically significant. These GC- and replicon-dependent variations in proportions of mutations mediated by large insertions were generally growth stage-independent (Fig. 2B and Fig. S2C). The evolved clones carrying large insertions in the PsacB-*sacB* region showed similar fitness under the selection condition (10% sucrose), as indicated by their growth curves (Fig. S2D).

### Replicon- and GC-dependent interactions

**Fig. 3:**
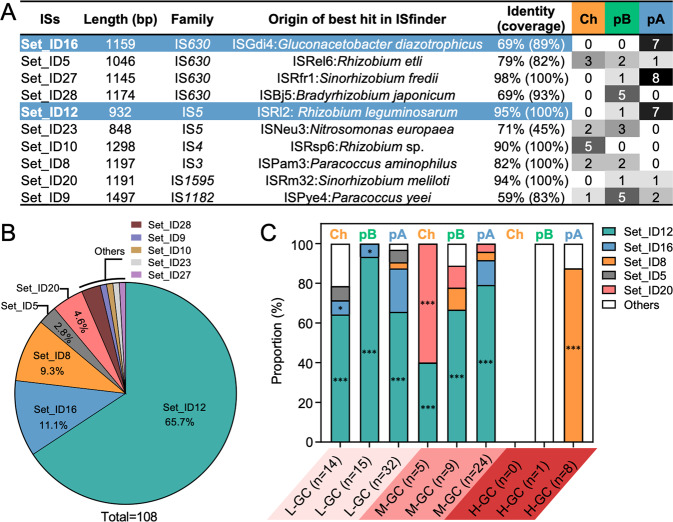
Replicon- and GC-dependent variation in insertion frequency of IS arsenal in the *sacB* gene. **A** Characteristics of the active ISs (the copy number of individual ISs in replicons of *S. fredii* CCBAU25509 is shown). **B** Pie chart of mutation events mediated by different active ISs. **C** GC- and replicon-dependent variation in insertion profiles of different active ISs. Significant enrichment of insertion events mediated by particular IS compared to its frequency among active ISs in the same replicon is indicated (*p < 0.05; ***p < 0.001; Fisher’s exact test). **A**–**C** Based on Sanger sequencing of 108 large insertion mutations within the coding region of *sacB*, collected at OD_600_ = 1.2 (Fig. 2).

Identities of putative transposable elements within the PsacB-*sacB* fragment were investigated for a total of 142 mutants collected from three independent representative pools at OD_600_ = 1.2 (Fig. 3). All large insertions were identified as mobile ISs ranging from 838 bp to 1497 bp and belonging to IS*630*, IS*5*, IS*4*, IS*3*, IS*1595*, and IS*1182* families (Fig. 3A; Table S2). There were 108 and 34 insertions in the coding region of *sacB* (Fig. 3B, C) and PsacB (Fig. S3), respectively. Set_ID12, an IS*5* family IS, accounted for 65.7% insertion mutation events within the *sacB* gene, which was followed by Set_ID16, Set_ID8, Set_ID5, Set_ID20, and five other ISs (Fig. 3B). Set_ID12 can insert into the coding region of L-GC and M-GC *sacB* localized in all replicons (Fig. 3C; significant enrichment compared to its frequency among active ISs in Ch, pB, and pA, *p* values < 0.001, Fisher’s exact test), though zero, one, and seven copies of Set_ID12 are present in Ch, pB, and pA replicons, respectively (Fig. 3A). All copies of the IS*3* family Set_ID8 are localized in Ch and pB, but Set_ID8 effectively inserted into H-GC *sacB* in pA rather than that in Ch or pB (Fig. 3C; significant enrichment in pA, *p* < 0.001, Fisher’s exact test). These results are in line with a working model that transposable ISs can freely move in the intracellular ecosystem, and there are notable replicon- and GC-dependent interactions between *sacB* and the IS community.

### Niche differentiation for transposable ISs in the multipartite genome ecosystem

**Fig. 4:**
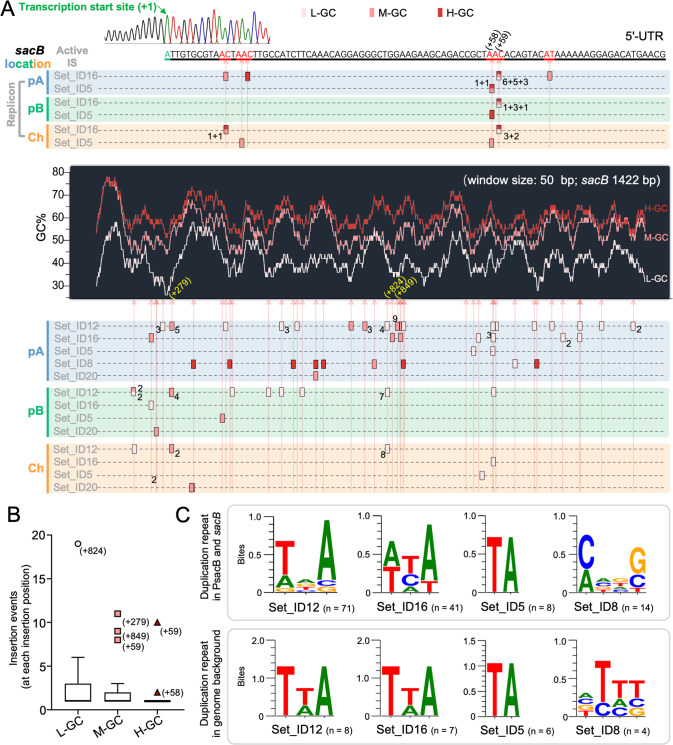
IS insertion positions in the 5′-UTR and *sacB* genes of different GC content. **A** Insertion sites in the 5′-UTR and associated *sacB* genes of different GC content (L-GC, M-GC, and H-GC) in the three replicons pSymA (pA), pSymB (pB), and chromosome (Ch). The number of independent clones carrying the same insertion event is indicated. **B** Box plot showing insertion hot spots within 5′-UTR and *sacB*. Insertion positions relative to the transcription start site are indicated in brackets for outliers (insertion hot spots) in (**A**) and (**B**). **C** Duplicated sequences due to insertions by corresponding ISs. Target sequence logos were generated by multiple sequence alignments of insertion sites within xenogeneic PsacB-*sacB* or genomic background using the program WebLogo. 142 mutants with large insertion mutation collected at OD_600_ = 1.2 (Fig. 2).

The transcription start site in PsacB was identified at the -88 nt position by 5′RACE (Fig. 4A), and all insertion events in the representative 142 mutants were within either the 88-nt 5′-UTR (34 mutants) or the 1422-nt coding region of *sacB* (108 mutants) (Fig. 4A). The insertion positions (relative to the transcription start site) of major active ISs are shown in Fig. 4A, and notable hot spots can be found for Set_ID12, Set_ID16, or Set_ID5, but not for Set_ID8 (Fig. 4A, B; outliers in the Box plot). The IS insertion events generate two directly repeated sequences as target duplication at the border during transposition [42]: T/A-N-A, A/T-H-A/T, and TA for Set_ID12, Set_ID16, and Set_ID5, respectively within PsacB-*sacB* (Fig. 4C; N = A/C/G/T; H = A/C/T; Table S2), which are comparable to their target sites T-T/A-A, T-T/A-A, and TA in the genome background of SF2 (Fig. 4C). By contrast, target sites seem to be more flexible for Set_ID8 (Fig. 4C).

**Fig. 5:**
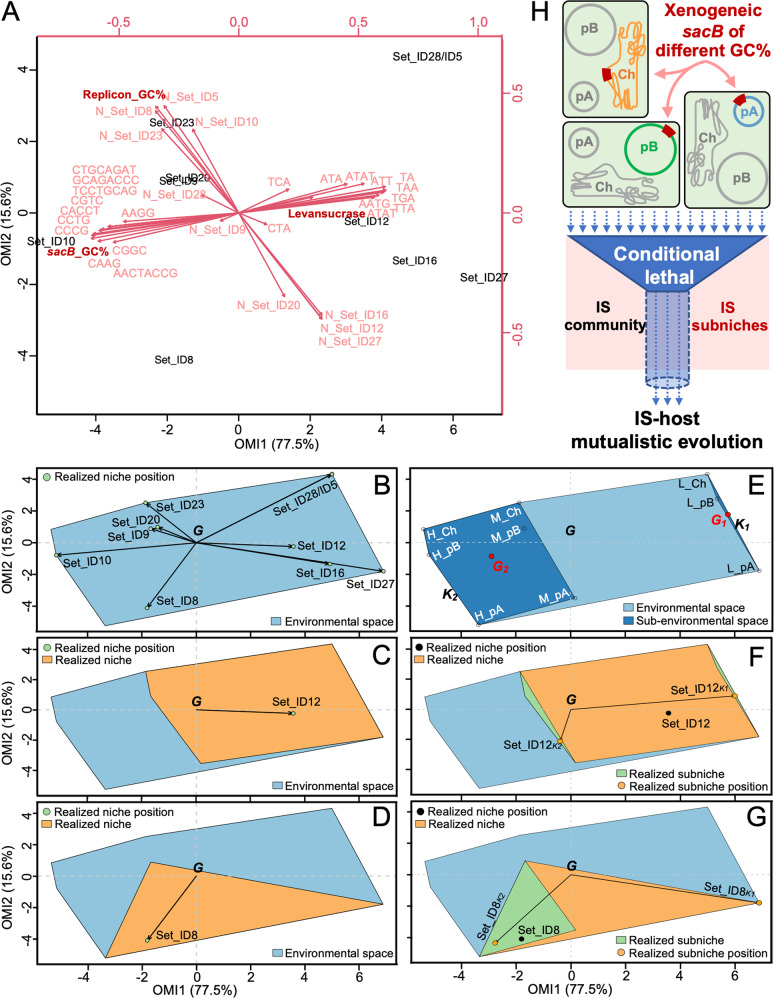
WitOMI analysis of niche differentiation for ISs during IS-host mutualistic evolution. **A** Canonical weights of environmental variables (red) and realized niche positions of active ISs (black). The first two OMI (outlying mean index) axes explained 93.1% of the total variability. N_Set_ID, number of each IS in the corresponding replicon where *sacB* is inserted. Nucleotide sequences, available insertion sites of ISs in different *sacB* versions. Levansucrase, SacB enzyme activity determined in Fig. 1C. **B** Realized niche positions of active ISs in the habitat conditions constraint of all *sacB* versions in the multipartite genome (environmental space). *G*, the average habitat conditions of the environmental space. Realized niche for Set_ID12 (**C**) and Set_ID8 (**D**). **E** Sub-environmental space *K*_*1*_ and *K*_*2*_. Nine colonizable sampling units (L_Ch, L_pB, L_pA; M_Ch, M_pB, M_pA; H_Ch, H_pB, H_pA) are indicated. *G*_*1*_ and *G*_*2*_ indicate the average subset habitat conditions found in *K*_*1*_ and *K*_*2*_, respectively. Realized subniche positions for Set_ID12 (**F**) and Set_ID8 (**G**) in *K*_*1*_ and *K*_*2*_. **H** Working model for the interaction between IS cocktail and realized subniches in the multipartite genome during IS-host mutualistic evolution.

In order to further uncover ecological factors shaping the insertion efficiency of individual ISs into the coding region of *sacB* (in nine common gardens: L_pA, L_pB, L_Ch, M_pA, M_pB, M_Ch, H_pA, H_pB, and H_Ch), *n*-dimensional ecological niches were characterized by within outlying mean index (WitOMI) analysis [40]. To define the environmental space, the copy number of individual ISs in each replicon (N_Set_ID), GC content of replicons (Replicon_GC%), GC content of *sacB* coding region (*sacB*_GC%), the levansucrase activity of strains carrying different *sacB* versions, and the number of available target sites identified for each IS (Table S3) in *sacB* genes were considered (Fig. 5A). The first two axes of the PCA explained 93.1% of the total variability (Fig. 5A). These ecological factors among nine colonizable common gardens collectively defined the environmental space and realized niches for the mobile active ISs (Fig. 5B–D and Fig. S4A). Set_ID12 and Set_ID16 but not Set_ID8 had marginal realized niche positions (Fig. 5B–D and Fig. S4A) which are significantly different from the mean environment conditions of the genomic environmental space (*G* in Fig. 5B–D and Fig. S4A; *p* values < 0.05, Monte Carlo test, 1000 permutations).

To get more insights into niche dynamics of active mobile ISs, the whole environmental space determined by nine test common gardens was further split up into two or three statistically analyzable subregions (sub-environmental space; *K*_*i*_ in Fig. 5E and Fig. S5A, subsets *p* values < 3.32E−42 and < 6.71E−16, respectively; Monte Carlo test, 1000 permutations) based on hierarchical clustering analysis of intracellular variables of nine sampling gardens. Notably, the subregion *K*_*1*_ harboring L_pA, L_pB, and L_Ch was consistently identified and has the most significant difference in environmental conditions compared to those of the whole environmental space (subsets *p* values < 2.76E−42). In the case of two subregions, *K*_*1*_ and *K*_*2*_ were delineated in a direction that’s tilted around 15 degrees from the OMI1 axis that explained 77.5% of the total variability (Fig. 5E). This space breakdown pattern was largely attributable to variations in the *sacB*_GC%, levansucrase activity, and available target sites identified for mobile ISs (variables *p* values < 0.05; except target sites TCA, ATA, ATAT, and AACTACCG) (Fig. 5A, E). The subregion *K*_*2*_ harboring the other common gardens (Fig. 5E) can be further delineated into one harboring M_pA and H_pA, and the other harboring M_pB, M_Ch, H_pB, and H_Ch (Fig. S5A) along a direction tilted around 30 degrees from the OMI2 axis that explained 15.6% of the total variability (Fig. 5E). This delineation was due to the variation of levansucrase activity (variable *p* < 0.05; Fig. 1C) and marginally significant difference in Replicon_GC% (variable *p* = 0.091) and in the copy number of Set_ID12 (*p* = 0.080), Set_ID16 (*p* = 0.091), Set_ID8 (*p* = 0.079), Set_ID23 (*p* = 0.072), and Set_ID27 (*p* = 0.091). Within these subregions and the whole environmental space, distinct but partially overlapping realized (sub)niches for the major active ISs (Set_ID12, Set_ID8, and Set_ID16) were identified (Fig. 5C–G, Fig. S4A, B and Fig. S5). Herein, realized subniche is used to describe delineation results of realized niche. Moreover, realized subniche positions for these three major active ISs within each subregion were uncovered (Fig. 5F–G, Fig. S4B and Fig. S5B–D) and are significantly different from the mean environmental conditions of the whole space (*G*; *p* values < 2.93E−16) and those of corresponding subregions (*G*_*i*_; *p* values < 2.67E−16). These results demonstrated distinct but overlapping (sub)niches for the major active ISs in the intracellular environmental space composed of nine colonizable common gardens. Therefore, the common garden approach established in this work experimentally revealed niche differentiation for IS community members during IS-host mutualistic evolution (Fig. 5H).

### Molecular evidence for niche differentiation for IS community

**Fig. 6:**
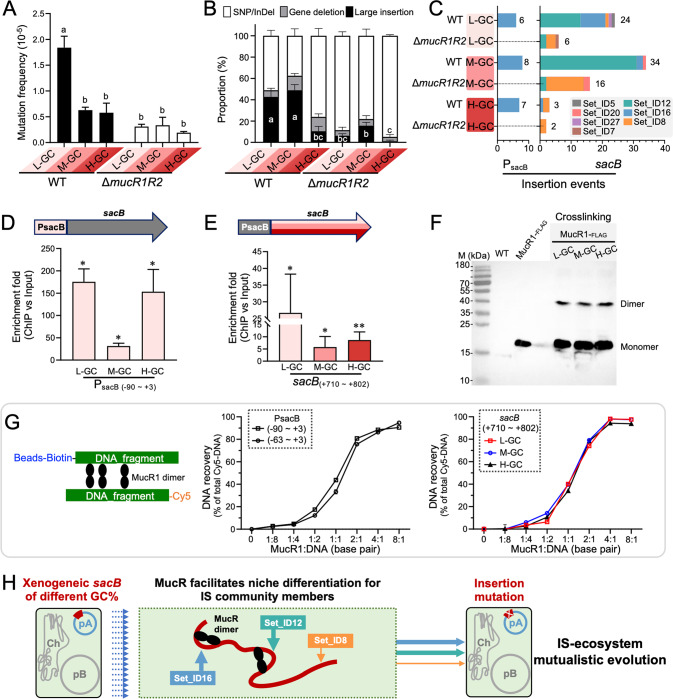
Xenogeneic silencer MucR facilitates niche differentiation for Set_ID16 and Set_ID12. **A** Total frequency of mutations conferring sucrose tolerance in WT and the Δ*mucR1R2* mutant harboring *sacB* of different GC% in pA. TY cultures of OD_600_ = 1.2 were used for mutant screening. **B** Proportions of mutation events mediated by large insertions, gene deletions, SNPs, and/or small InDels. **A**, **B** Three independent experiments were performed. Different letters indicate significant difference (Average ± SEM; ANOVA followed by Duncan’s test, alpha = 0.05) between mutation frequency values (**A**) or between proportion values for large insertion events (**B**). **C** Insertion frequency of Set_ID16 and Set_ID12 in PsacB and *sacB* was reduced in the Δ*mucR1R2* mutant. ChIP-qPCR showing that MucR1 binds PsacB (**D**) and *sacB* fragments (**E**) in vivo. *p < 0.05; **p < 0.01; significant difference based on one-sample t-test. **F** Crosslinking assay of MucR1-FLAG in WT carrying *sacB* of different GC% in pA (1% formaldehyde for 15 min). **G** Microscale thermophoresis showing that MucR1 can form DNA-MucR1-DNA bridging complex in two PsacB fragments and a *sacB* region of different GC% in vitro (Average ± SEM based on four technical replicates). **H** Working model of niche differentiation for IS community members mediated by MucR during IS-ecosystem mutualistic evolution.

It is clear that major active ISs including Set_ID12, Set_ID16, and Set_ID8 are more likely to insert into xenogeneic PsacB-*sacB* in the pA replicon (Fig. 3C, Fig. S3B, Fig. 4A, Fig. 5, Fig. S4 and Fig. S5). The pA replicon is enriched with periodic Ts bound by the xenogeneic silencer MucR in *S. fredii* [29], and another convergent well-known xenogeneic silencer H-NS promotes transposition of various transposable elements such as Tn*10*, Tn*5*, Tn*552*, and IS*903* in *Gammaproteobacteria* [43–46]. SF2 has two *mucR* copies with *mucR1* and *mucR2* in the Ch and pA replicons, respectively. They are 100% identical to their homologs characterized previously in *S. fredii* CCBAU45436 [29, 47]. Here we further investigated to what extent the interactions between IS community and xenogeneic PsacB-*sacB* can be modulated by xenogeneic silencer MucR. To this end, L-, M-, and H-GC *sacB* together with PsacB were inserted into the pA replicon of the Δ*mucR1R2* mutant (Table S1), and PsacB-*sacB* mutants were screened as described previously in the presence of 10% sucrose with the corresponding WT derivatives as controls. Three resultant Δ*mucR1R2* derivatives had lower mutation frequency in the PsacB-*sacB* fragment compared to the corresponding WT derivatives (Fig. 6A, statistically significant for the L-GC pair). Notably, the proportion of mutation events mediated by the IS community was significantly reduced in Δ*mucR1R2* derivatives harboring L- and M-GC *sacB* genes (Fig. 6B), which was largely attributable to the absence of Set_ID16 in the PsacB-*sacB* fragment and significant reduction of Set_ID12 by 91% in the *sacB* gene in the Δ*mucR1R2* mutant (Fig. 6C). By contrast, the number of insertion mutations mediated by Set_ID8 was increased in the *sacB* gene in the Δ*mucR1R2* mutant (Fig. 6C). These contrasting insertion efficiencies among IS community members were not explainable by their transcriptional levels in either WT or the Δ*mucR1R2* mutant background (Fig. S6). In short, these results implied distinct niche positions preferred by Set_ID12 and Set_ID16 along the PsacB-*sacB* fragment (also revealed in Fig. 4A). Particularly, these ecological processes depended on a functional MucR.

Cumulating evidence suggests that xenogeneic silencer H-NS promotes transposition of various mobile elements in different ways [43–46], though diverse nucleoprotein complexes (known as transpososomes) acting in transposition processes involve bent target DNA that can be generated in the DNA-H-NS-DNA bridging complex [48, 49]. Indeed, ChIP-qPCR analysis revealed that convergently evolved xenogeneic silencer MucR1 can be recruited to the 5′-UTR region in vivo (Fig. 6D) where Set_ID16 was the major active IS (Fig. 6C), and to multiple regions within the *sacB* gene (Fig. 6E and Fig. S7A) where multiple ISs members were involved and with Set_ID12 as the major active IS (Fig. 6C). Moreover, MucR1 can form dimers in the WT derivatives carrying *sacB* of different GC% in the pA replicon (Fig. 6F), and establish DNA-MucR1-DNA bridging complexes with individual DNA target fragments from the PsacB region or *sacB* coding region of different GC content in vitro (Fig. 6G and Fig. S7B). Despite similar duplicated direct repeat sequences generated after transposition of Set_ID16 and Set_ID12 (Fig. 4B), the 10-bp flanking regions of their insertion positions showed distinct sequence features regarding the average GC% difference between up- and downstream sequences (Fig. S8; 4% vs 18% for Set_ID16 vs Set_ID12). Taken together, the convergently evolved xenogeneic silencer MucR facilitated insertion of Set_ID16 and Set_ID12 into their distinct differentiated niches at least partially by forming DNA-MucR-DNA bridging complexes (Fig. 6H). Experimental elimination of this process led to a downshift in insertion efficiency of Set_ID12 and Set_ID16 but upshift of Set_ID8 insertion frequency. This phenomenon may be mediated by MucR-dependent DNA topology changes which facilitated insertion by Set_ID12 and Set_ID16 while limiting insertion sites available for Set_ID8. This MucR-dependent niche differentiation process is also supported by the notably higher GC% of flanking regions of Set_ID8 insertion sites in *sacB* compared to those of Set_ID12 and Set_ID16 (Fig. S8).

## Discussion

This work was focused on bacterial ISs which show considerable interspecific variations in distribution and abundance [37, 50]. This variation pattern has been tentatively explained by a niche-restricted bacterial evolution model whereby, in the evolutionary timescale, IS expansion occurs in some extremophiles (e.g. some cyanobacteria and *Sulfolobus solfataricus*) and bacteria facultatively interacting with nutritionally rich eukaryote hosts while IS elimination accompanies the multi-step genome streamlining process during the evolution of obligate microsymbionts [51]. In silico modeling suggests that the long-term fate of TEs in asexual microorganisms is extinction and opportunity for TE persistence depends on horizontal gene transfer [52, 53]. Indeed, ISs are particularly enriched in horizontally transferable plasmids or genomic islands in bacteria such as those in rhizobia facultatively associated with diverse legumes [26, 27, 54, 55]. IS accumulation will also be facilitated if IS transpositions have beneficial effects, which have been recurrently reported in bacterial adaptive evolution experiments [27, 56, 57]. Moreover, neutral or deleterious insertions may also accumulate due to genetic drift or frequent horizontal transfer between hosts [10, 24, 58–60]. It should be noted that pioneer studies of TE ecology have been restricted to eukaryotes to our knowledge [10, 24, 58–60]. It is hypothesized that the observed variation in distribution and abundance of TE families is mainly modulated by evolutionary processes while that of individual TEs between closely related genomes may be largely explained by ecological processes [16]. TE insertion specificity levels and variations in genomic features are expected to be underlying TE ecology within a eukaryote genome [17, 25]. Various aspects of TE ecology have been partially tested by descriptive statistical analyses and idealized theoretical simulations in eukaryotes [17–25, 58, 61]. These efforts have uncovered intriguing correlational explanations which are however sometimes controversial and have not been effectively tested by experiments [24, 58, 61]. The reason is either due to the focused evolutionary processes beyond the experimental timescale [51] or without a well-controlled testable ecosystem.

This work experimentally investigated causal ecological interactions that determine distribution and abundance of ISs within bacterial cells, which can be considered as ecology at the intracellular level. The common garden approach is generally used to identify causal relationships underlying geographic variation of organisms in ecology [15], which is however not established in genome ecology. Similar to most cellular organisms [16, 25], SF2 has a biased distribution of ISs in its multipartite genome (Fig. 3A) [27]. The distribution and abundance of individual ISs correlated with GC content of replicons in an IS-dependent manner (Fig. 5A, along OMI2). This in situ correlation relationship was experimentally tested by introducing three xenogeneic *sacB* genes of different GC content in their synonymous codons into three replicons, resulting nine common gardens. Indeed, GC content and available target sites of *sacB* genes were identified as two major causal factors determining the transposition efficiency for different mobile ISs in the IS-ecosystem mutualistic evolution experiment (Fig. 5A, along OMI1). Moreover, ISs with biased in situ distribution and abundance in the multipartite genome (Fig. 5A, along OMI2) can insert into their preferred target sites of *sacB* across three replicons, suggesting niche differentiation (Fig. 3C, Fig. 4 and Fig. 5). This strengthens the view that mobile ISs are useful analogs of organisms in genome ecology [10]. The intracellular common garden approach established herein can be completed within two weeks (Fig. 1), and can be further explored to test general laws of ecology such as the metapopulation theory involving dispersal between colonizable patches [62].

The IS-ecosystem mutualistic evolution experiment in the context of nine common gardens and within outlying mean index analysis [40] also allowed us to identify partially overlapping niches of the major active ISs in the environmental space of this multipartite genome (Fig. 4, Fig. 5, Fig. S4 and Fig. S5). Set_ID12, Set_ID16, and Set_ID8 were more likely to be captured by their corresponding preferred baits in the pA replicon than in the other two replicons (Fig. 5E–G, Fig. S4 and Fig. S5, along OMI2). This biased transposition pattern can be partially explained by replicon-associated factors including Replicon_GC% and the copy number of active ISs in each replicon (along OMI2 in Fig. 5, Fig. S4 and Fig. S5), though to a lesser extent than the sequence signatures of *sacB* (along OMI1 in Fig. 5, Fig. S4 and Fig. S5). The pA replicon is characterized by the lowest GC content among the three replicons, and it is enriched with AT-rich accessory genes directly targeted by the xenogeneic silencer MucR [26, 29]. We demonstrated that MucR can form a DNA-MucR-DNA bridging complex in multiple regions of xenogeneic PsacB-*sacB* fragments and facilitate these baits to capture Set_ID12 and Set_ID16 (Fig. 6). This is consistent with earlier studies on the convergently evolved xenogeneic silencer H-NS in *Gammaproteobacteria*, which promotes transposition of several TEs [43–46, 63] at least partially by forming DNA-H-NS-DNA bridging complex [48, 49]. Moreover, the common garden approach in the *mucR* deletion mutant uncovered a working model of indirect resource exploitation competition between active ISs likely mediated by MucR-dependent DNA topology changes (Fig. 6). This may represent an intriguing interspecific competition phenomenon in genome ecology, which involves not only TEs but also another “biotic factor” that positively or negatively affects the transposition of IS community members. The MucR-dependent insertion efficiency of major active ISs into the conditional deleterious gene (Fig. 6) was also supported by the evidence (Fig. S9) that the Δ*mucR1R2* mutant of SF2 failed to evolve into compatible microsymbionts of the same commercial soybean cultivar used earlier [27]. Therefore this work built an efficient experimental paradigm to study largely unexplored causal relationships underlying the distribution and abundance of mobile ISs [10, 12, 17, 25].

**Fig. 7:**
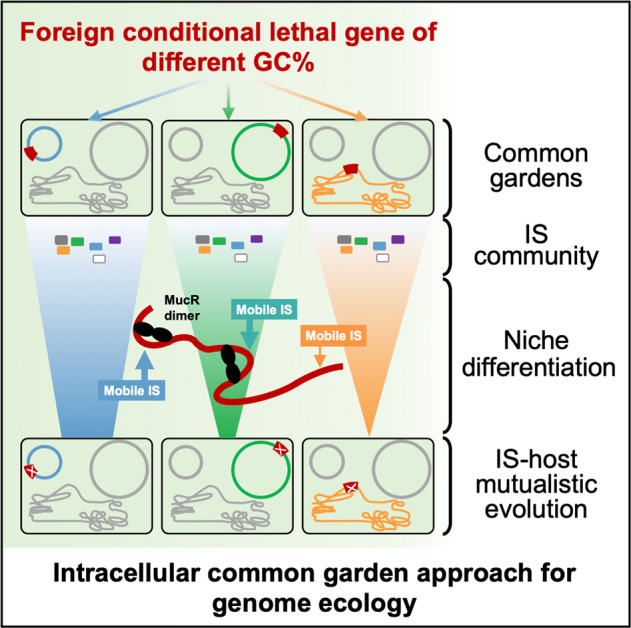
An intracellular common garden approach for genome ecology. Common gardens (red foreign genes) in different replicons, IS community members (filled rectangles), and IS niche differentiation facilitated by the convergently evolved xenogeneic silencer MucR (filled black ellipses) are indicated, during adaptive evolution of rhizobia carrying foreign conditional lethal genes of different sequence signatures.

The intracellular common garden system established in this study (Fig. 7) can be further modified to test other causal relationships in genome ecology by artificially manipulating various intracellular variables within the well-bounded bacterial cellular ecosystems. This is in contrast to the fuzzy boundary of canonical ecosystems [10, 12, 17]. Moreover, we are not at a stage when main ecological questions have been solved [15], and it is likely that advances in intracellular genome ecology may provide helpful insight into universal ecological principles [10]. Among diverse cellular organisms, bacteria are characterized by short generation times and have been intensively studied using experimental evolution methods to understand adaptions under various controlled conditions [64]. Therefore, working with bacterial cellular ecosystems allows addressing the ultimate evolutionary explanation for an observed genome ecology pattern in laboratory time as demonstrated in this work. On the other hand, bacterial cellular ecosystems are well-bounded but not completely isolated from biotic and abiotic environmental factors. Natural horizontal gene transfer and synthetic biology can bring novel parts and circuits into chasis cellular ecosystems [65, 66]. As highlighted by the replicon- and GC-dependent variation in genetic stability of xenogeneic *sacB* genes of different GC content, the intracellular common garden approach can provide novel insight into genetic stability of focused genome regions from the dimension of emerging genome ecology. Given the rapid development of synthetic biology and systems biology, advances in intracellular genome ecology in the well-bounded cellular ecosystems can support the updating from conventional benchwork-based molecular biology to theory-based molecular biology.

## Supplementary information

Figure S1

Figure S2

Figure S3

Figure S4

Figure S5

Figure S6

Figure S7

Figure S8

Figure S9

Table S1

Table S2

Table S3

## Data Availability

All data generated or analyzed during this study are included in this published article and its supplementary information files. Genome sequence of *S. fredii* CCBAU25509 is available from the NCBI database (BioSample: SAMN03761947).

## References

[1] McClintock B (1950). The origin and behavior of mutable loci in maize. Proc Natl Acad Sci USA.

[2] Colonna Romano N, Fanti L (2022). Transposable elements: major players in shaping genomic and evolutionary patterns. Cells.

[3] Bourque G, Burns KH, Gehring M, Gorbunova V, Seluanov A, Hammell M (2018). Ten things you should know about transposable elements. Genome Biol.

[4] Blommaert J (2020). Genome size evolution: towards new model systems for old questions. Proc R Soc B Biol Sci.

[5] Biemont C (2010). A brief history of the status of transposable elements: from junk DNA to major players in evolution. Genetics.

[6] Faure G, Shmakov SA, Yan WX, Cheng DR, Scott DA, Peters JE (2019). CRISPR–Cas in mobile genetic elements: counter-defence and beyond. Nat Rev Microbiol.

[7] Hickman AB, Dyda F (2016). DNA transposition at work. Chem Rev.

[8] Altae-Tran H, Kannan S, Demircioglu FE, Oshiro R, Nety SP, McKay LJ (2021). The widespread IS200/IS605 transposon family encodes diverse programmable RNA-guided endonucleases. Science.

[9] Karvelis T, Druteika G, Bigelyte G, Budre K, Zedaveinyte R, Silanskas A (2021). Transposon-associated TnpB is a programmable RNA-guided DNA endonuclease. Nature.

[10] Kidwell MG, Lisch DR (2001). Perspective: transposable elements, parasitic DNA, and genome evolution. Evolution.

[11] Kidwell MG, Lisch D (1997). Transposable elements as sources of variation in animals and plants. Proc Natl Acad Sci USA.

[12] Brookfield JFY (2005). The ecology of the genome-mobile DNA elements and their hosts. Nat Rev Genet.

[13] Le Rouzic A, Dupas S, Capy P (2007). Genome ecosystem and transposable elements species. Gene.

[14] Venner S, Feschotte C, Biémont C (2009). Dynamics of transposable elements: towards a community ecology of the genome. Trends Genet.

[15] Begon M, Townsend CR. Ecology: from individuals to ecosystems, 5th ed. Hoboken, New Jersey, USA:Wiley; 2021.

[16] Linquist S, Saylor B, Cottenie K, Elliott TA, Kremer SC, Gregory TR (2013). Distinguishing ecological from evolutionary approaches to transposable elements. Biol Rev.

[17] Saylor B, Elliott TA, Linquist S, Kremer SC, Gregory TR, Cottenie K (2013). A novel application of ecological analyses to assess transposable element distributions in the genome of the domestic cow, *Bos taurus*. Genome.

[18] Promislow DEL, Jordan IK, McDonald JF (1999). Genomic demography: a life-history analysis of transposable element evolution. Proc R Soc B Biol Sci.

[19] Venner S, Miele V, Terzian C, Biémont C, Daubin V, Feschotte C (2017). Ecological networks to unravel the routes to horizontal transposon transfers. PLoS Biol.

[20] Bourgeois Y, Ruggiero RP, Hariyani I, Boissinot S (2020). Disentangling the determinants of transposable elements dynamics in vertebrate genomes using empirical evidences and simulations. PLoS Genet.

[21] Abrusán G, Krambeck HJ (2006). Competition may determine the diversity of transposable elements. Theor Popul Biol.

[22] Pavlov S, Gursky VV, Samsonova M, Kanapin A, Samsonova A (2021). Stochastic effects in retrotransposon dynamics revealed by modeling under competition for cellular resources. Life.

[23] Xue C, Goldenfeld N (2016). Stochastic predator-prey dynamics of transposons in the human genome. Phys Rev Lett.

[24] Linquist S, Cottenie K, Elliott TA, Saylor B (2015). Applying ecological models to communities of genetic elements: the case of neutral theory. Mol Ecol.

[25] Stitzer MC, Anderson SN, Springer NM, RossIbarra J (2021). The genomic ecosystem of transposable elements in maize. PLoS Genet.

[26] Jiao J, Ni M, Zhang B, Zhang Z, Young JPW, Chan T-F (2018). Coordinated regulation of core and accessory genes in the multipartite genome of *Sinorhizobium fredii*. PLoS Genet.

[27] Zhao R, Liu LX, Zhang YZ, Jiao J, Cui WJ, Zhang B (2018). Adaptive evolution of rhizobial symbiotic compatibility mediated by co-evolved insertion sequences. ISME J.

[28] Cui W, Zhang B, Zhao R, Liu L, Jiao J, Zhang Z (2021). Lineage-specific rewiring of core pathways predating innovation of legume nodules shapes symbiotic efficiency. mSystems.

[29] Jiao J, Zhang B, Li M-L, Zhang Z, Tian C-F (2022). The zinc-finger bearing xenogeneic silencer MucR in α-proteobacteria balances adaptation and regulatory integrity. ISME J.

[30] Shi W-T, Zhang B, Li M-L, Liu K-H, Jiao J, Tian C-F (2022). The convergent xenogeneic silencer MucR predisposes α-proteobacteria to integrate AT-rich symbiosis genes. Nucleic Acids Res.

[31] Harrison PW, Lower RPJ, Kim NKD, Young JPW (2010). Introducing the bacterial ‘chromid’: not a chromosome, not a plasmid. Trends Microbiol.

[32] Quandt J, Hynes MF (1993). Versatile suicide vectors which allow direct selection for gene replacement in Gram-negative bacteria. Gene.

[33] Kovach ME, Elzer PH, Hill DS, Robertson GT, Farris MA, Roop RM (1995). Four new derivatives of the broad-host-range cloning vector pBBR1MCS, carrying different antibiotic-resistance cassettes. Gene.

[34] Hu Y, Jiao J, Liu LX, Sun YW, Chen W, Sui X (2018). Evidence for phosphate starvation of rhizobia without terminal differentiation in legume nodules. Mol Plant-Microbe Interact.

[35] Ditta G, Stanfield S, Corbin D, Helinski DR (1980). Broad host range DNA cloning system for gram-negative bacteria: construction of a gene bank of *Rhizobium meliloti*. Proc Natl Acad Sci USA.

[36] van der Valk RA, Vreede J, Qin L, Moolenaar GF, Hofmann A, Goosen N (2017). Mechanism of environmentally driven conformational changes that modulate H-NS DNA-Bridging activity. Elife.

[37] Varani AM, Siguier P, Gourbeyre E, Charneau V, Chandler M (2011). ISsaga is an ensemble of web-based methods for high throughput identification and semi-automatic annotation of insertion sequences in prokaryotic genomes. Genome Biol.

[38] Crooks GE, Hon G, Chandonia JM, Brenner SE (2004). WebLogo: a sequence logo generator. Genome Res.

[39] Peterson AT (2011). Ecological niche conservatism: a time-structured review of evidence. J Biogeogr.

[40] Karasiewicz S, Dolédec S, Lefebvre S (2017). Within outlying mean indexes: refining the OMI analysis for the realized niche decomposition. PeerJ.

[41] Karasiewicz S. Subniche documentation for the within outlying mean indexes calculations (WitOMI). https://github.com/KarasiewiczStephane/WitOMI.

[42] Siguier P, Gourbeyre E, Varani A, Ton-Hoang B, Chandler M. Everyman’s guide to bacterial insertion sequences. Microbiol Spectr. 2015;3:MDNA3-0030–2014. 10.1128/microbiolspec.MDNA3-0030-2014 26104715

[43] Ward CM, Wardle SJ, Singh RK, Haniford DB (2007). The global regulator H-NS binds to two distinct classes of sites within the Tn10 transpososome to promote transposition. Mol Microbiol.

[44] Wardle SJ, Chan A, Haniford DB (2009). H-NS binds with high affinity to the Tn10 transpososome and promotes transpososome stabilization. Nucleic Acids Res.

[45] Whitfield CR, Wardle SJ, Haniford DB (2009). The global bacterial regulator H-NS promotes transpososome formation and transposition in the Tn5 system. Nucleic Acids Res.

[46] Swingle B, O’Carroll M, Haniford D, Derbyshire KM (2004). The effect of host-encoded nucleoid proteins on transposition: H-NS influences targeting of both IS903 and Tn10. Mol Microbiol.

[47] Jiao J, Wu LJ, Zhang B, Hu Y, Li Y, Zhang XX (2016). MucR is required for transcriptional activation of conserved ion transporters to support nitrogen fixation of *Sinorhizobium fredii* in soybean nodules. Mol Plant-Microbe Interact.

[48] Qin L, Erkelens AM, Ben Bdira F, Dame RT (2019). The architects of bacterial DNA bridges: a structurally and functionally conserved family of proteins. Open Biol.

[49] Dyda F, Chandler M, Hickman AB (2012). The emerging diversity of transpososome architectures. Q Rev Biophys.

[50] Siguier P, Perochon J, Lestrade L, Mahillon J, Chandler M (2006). ISfinder: the reference centre for bacterial insertion sequences. Nucleic Acids Res.

[51] Siguier P, Gourbeyre E, Chandler M (2014). Bacterial insertion sequences: their genomic impact and diversity. FEMS Microbiol Rev.

[52] Park HJ, Gokhale CS, Bertels F (2021). How sequence populations persist inside bacterial genomes. Genetics.

[53] Van Dijk B, Bertels F, Stolk L, Takeuchi N, Rainey PB (2022). Transposable elements promote the evolution of genome streamlining. Philos Trans R Soc B Biol Sci.

[54] diCenzo GC, Finan TM (2017). The divided bacterial genome: structure, function, and evolution. Microbiol Mol Biol Rev.

[55] Pistorio M, Giusti MA, Del Papa MF, Draghi WO, Lozano MJ, Torres Tejerizo G (2008). Conjugal properties of the *Sinorhizobium meliloti* plasmid mobilome. FEMS Microbiol Ecol.

[56] Sugawara M, Takahashi S, Umehara Y, Iwano H, Tsurumaru H, Odake H (2018). Variation in bradyrhizobial NopP effector determines symbiotic incompatibility with Rj2-soybeans via effector-triggered immunity. Nat Commun.

[57] Consuegra J, Gaffé J, Lenski RE, Hindré T, Barrick JE, Tenaillon O (2021). Insertion-sequence-mediated mutations both promote and constrain evolvability during a long-term experiment with bacteria. Nat Commun.

[58] Kremer SC, Linquist S, Saylor B, Elliott TA, Gregory TR, Cottenie K (2020). Transposable element persistence via potential genome-level ecosystem engineering. BMC Genomics.

[59] Doolittle WF (2022). All about levels: transposable elements as selfish DNAs and drivers of evolution. Biol Philos.

[60] Lynch M, Bobay LM, Catania F, Gout JF, Rho M (2011). The repatterning of eukaryotic genomes by random genetic drift. Annu Rev Genomics Hum Genet.

[61] Butler CL, Bell EA, Taylor MI (2021). Removal of beneficial insertion effects prevent the long-term persistence of transposable elements within simulated asexual populations. BMC Genomics.

[62] Ovaskainen O, Saastamoinen M (2018). Frontiers in metapopulation biology: the legacy of Ilkka Hanski. Annu Rev Ecol Evol Syst.

[63] Jiao J, Tian C-F (2020). Ancestral zinc-finger bearing protein MucR in alpha-proteobacteria: a novel xenogeneic silencer?. Comput Struct Biotechnol J.

[64] Payne JL, Wagner A (2019). The causes of evolvability and their evolution. Nat Rev Genet.

[65] Whelan FJ, Hall RJ, McInerney JO (2021). Evidence for selection in the abundant accessory gene content of a prokaryote pangenome. Mol Biol Evol.

[66] Cameron DE, Bashor CJ, Collins JJ (2014). A brief history of synthetic biology. Nat Rev Microbiol.

