# Supplementary material for: Intracellular common gardens reveal niche differentiation in transposable element community during bacterial adaptive evolution
Source: ISME J. 2022 Nov 24;17(2):297–308. doi: 10.1038/s41396-022-01344-2 (PMC9860058; doi:10.1038/s41396-022-01344-2)
Supplement: Supplementary file 1 — Figure S1 [file 41396_2022_1344_MOESM1_ESM.pdf]

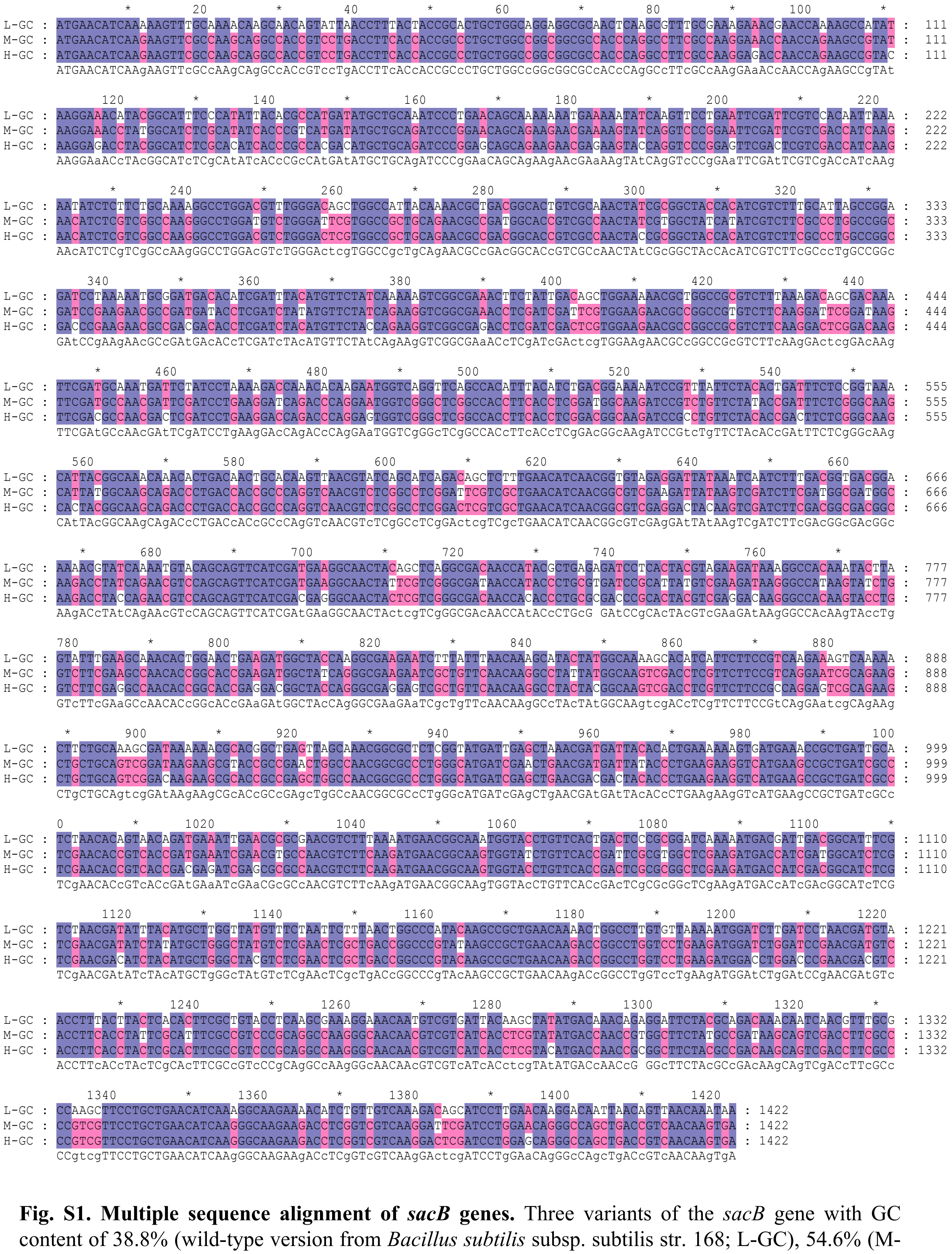

Fig. S1. Multiple sequence alignment of *sacB* genes. Three variants of the *sacB* gene with GC content of 38.8% (wild-type version from *Bacillus subtilis* subsp. subtilis str. 168; L-GC), 54.6% (M-GC), and 61.6% (H-GC).
