# Supplementary material for: Intracellular common gardens reveal niche differentiation in transposable element community during bacterial adaptive evolution
Source: ISME J. 2022 Nov 24;17(2):297–308. doi: 10.1038/s41396-022-01344-2 (PMC9860058; doi:10.1038/s41396-022-01344-2)
Supplement: Supplementary file 2 — Figure S2 [file 41396_2022_1344_MOESM2_ESM.pdf]

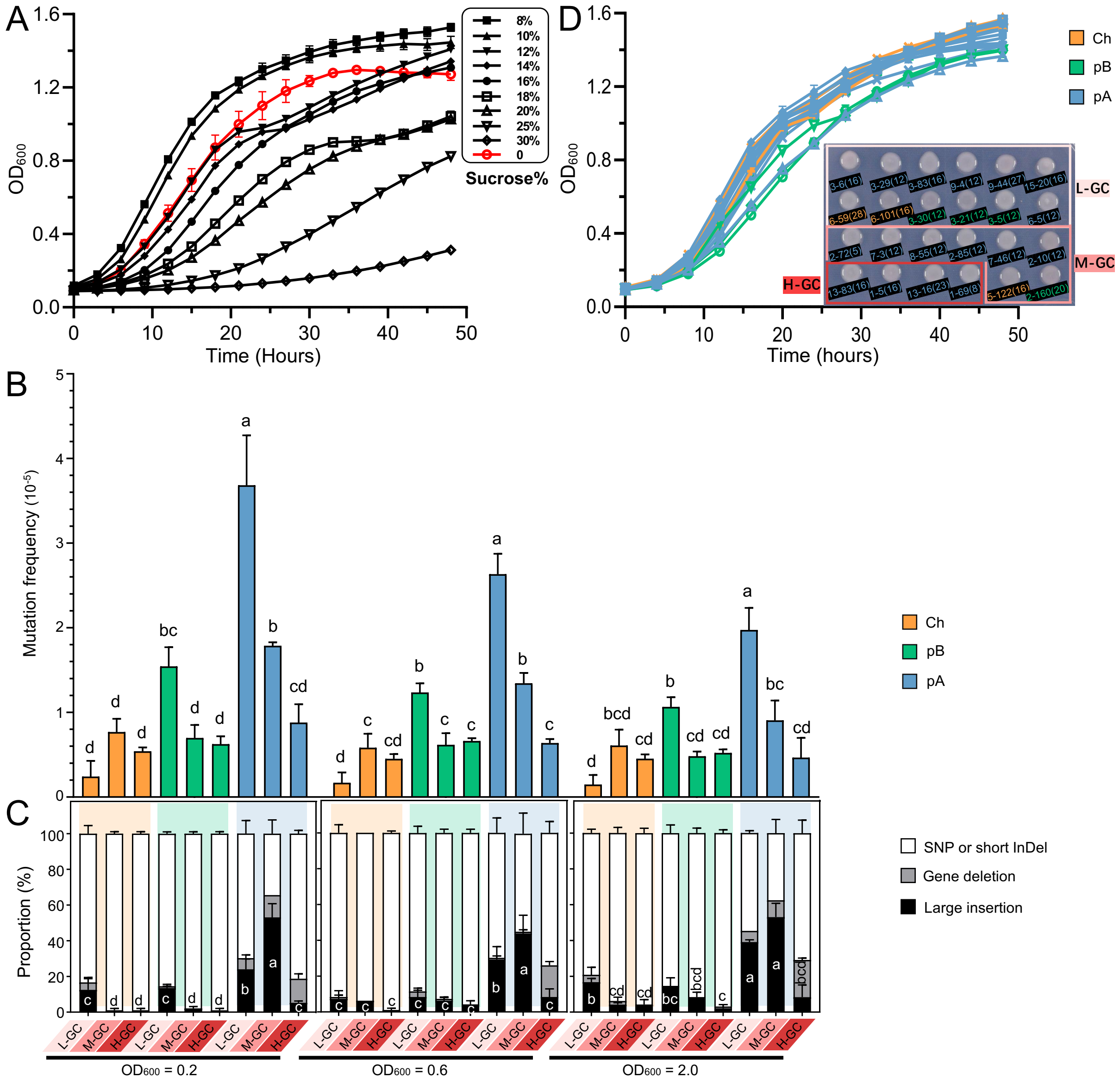

Fig. S2. Growth curves of SF2 and summary of adaptive mutations in SF2 derivatives carrying PsacB-*sacB* under 10% sucrose. (**A**) Growth curves of SF2 in the TY medium supplemented with different concentrations of sucrose. Results are based on average ± SEM of three biological replicates. (**B**) Total mutation frequency of PsacB-*sacB* in log and stationary phases. (**C**) Proportions of mutation events mediated by different mutation mechanisms: large insertions, gene deletions, SNPs and/or small InDels. Three independent experiments were performed. Different letters indicate significant difference (Average ± SEM; ANOVA followed by Duncan's test, alpha = 0.05) between mutation frequency values (**B**) or between proportion values for large insertion events (**C**). (**D**) Growth curves and 3-day colonies of representative evolved clones carrying IS in the PsacB-*sacB* region (TY medium containing 10% sucrose). Mutant IDs (see Table S2 for details) and their active ISs (Set_ID numbers in brackets) are shown on the colony picture. Mutants evolved from SF2 derivatives carrying *sacB* of L-GC, M-GC, and H-GC in each replicon are indicated by different colors in (**C**) and (**D**).
