# Supplementary material for: Intracellular common gardens reveal niche differentiation in transposable element community during bacterial adaptive evolution
Source: ISME J. 2022 Nov 24;17(2):297–308. doi: 10.1038/s41396-022-01344-2 (PMC9860058; doi:10.1038/s41396-022-01344-2)
Supplement: Supplementary file 3 — Figure S3 [file 41396_2022_1344_MOESM3_ESM.pdf]

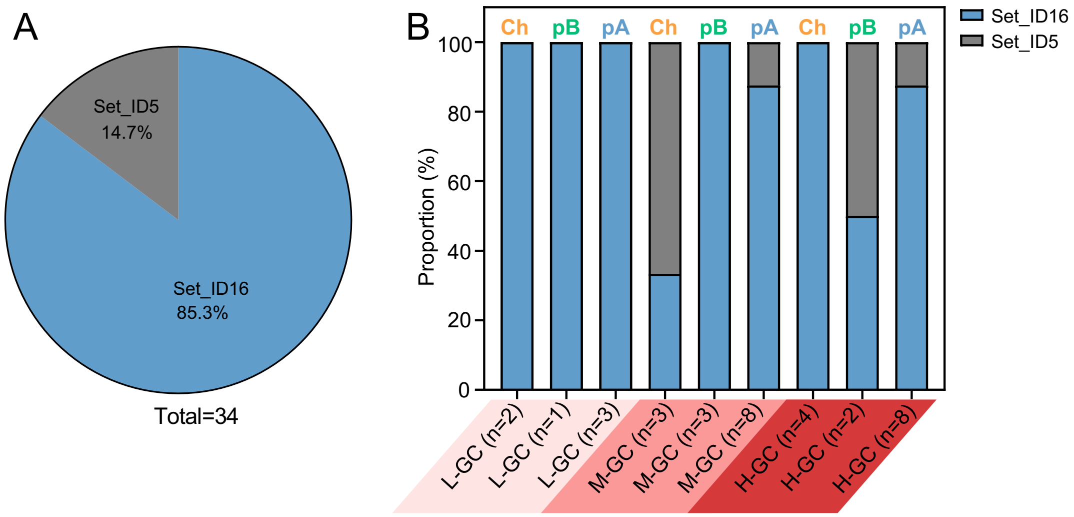

Fig. S3. Replicon- and GC-dependent variation in insertion frequency of IS arsenal in PsacB. (**A**) Pie chart of mutation events mediated by different active ISs. (**B**) GC- and replicon-dependent variation in insertion profiles of different active ISs. Based on Sanger sequencing and subsequent IS identification for 142 mutants with large insertion mutation collected at OD_{600} = 1.2 (Fig 2), and 34 insertions found within PsacB were analyzed herein.
