# Supplementary material for: Intracellular common gardens reveal niche differentiation in transposable element community during bacterial adaptive evolution
Source: ISME J. 2022 Nov 24;17(2):297–308. doi: 10.1038/s41396-022-01344-2 (PMC9860058; doi:10.1038/s41396-022-01344-2)
Supplement: Supplementary file 4 — Figure S4 [file 41396_2022_1344_MOESM4_ESM.pdf]

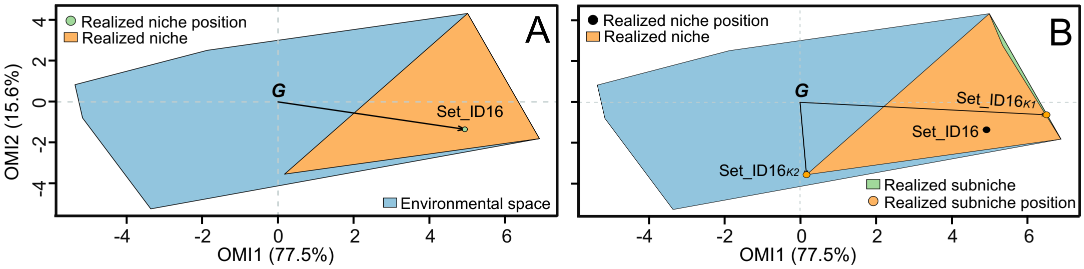


**Fig. S4. Within outlying mean index analysis of niche differentiation for Set_ID16.** (**A**) Realized niche for Set_ID16. (**B**) Realized subniche for Set_ID16 in two subsets. The first two OMI (outlying mean index) axes explained 93.1% of the total variability.
