# Supplementary material for: Intracellular common gardens reveal niche differentiation in transposable element community during bacterial adaptive evolution
Source: ISME J. 2022 Nov 24;17(2):297–308. doi: 10.1038/s41396-022-01344-2 (PMC9860058; doi:10.1038/s41396-022-01344-2)
Supplement: Supplementary file 5 — Figure S5 [file 41396_2022_1344_MOESM5_ESM.pdf]

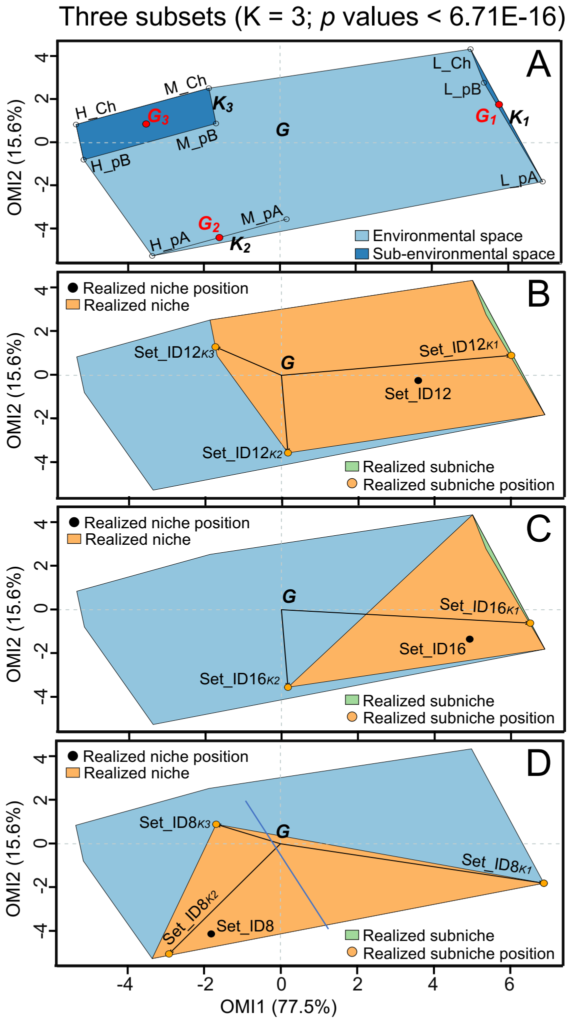

Fig. S5. Within outlying mean index analysis of niche differentiation for ISs for three subsets. (**A**) Three sub-environmental spaces are indicated by K_i (i = 1, 2, 3) in three subsets, respectively. Subsets *P* values based on Monte Carlo test with 1000 permutations are shown. The average subset habitat conditions found in K_i are indicated by G_i. Nine colonizable sampling unites (L_Ch, L_pB, L_pA; M_Ch, M_pB, M_pA; H_Ch, H_pB, H_pA) are shown. (**B-D**) Realized subniche positions for Set_ID12 (**B**), Set_ID16 (**C**), and Set_ID8 (**D**) are indicated by Set_ID$_{Ki}$ in the corresponding sub-environmental space K_i. Fig S5C is the same to Fig S4B due to the absence of Set_ID16 in the subregion K_3. Realized niche positions of each IS in the whole data set are also indicated by black point. The first two OMI (outlying mean index) axes explained 93.1% of the total variability.
