# Supplementary material for: Intracellular common gardens reveal niche differentiation in transposable element community during bacterial adaptive evolution
Source: ISME J. 2022 Nov 24;17(2):297–308. doi: 10.1038/s41396-022-01344-2 (PMC9860058; doi:10.1038/s41396-022-01344-2)
Supplement: Supplementary file 6 — Figure S6 [file 41396_2022_1344_MOESM6_ESM.pdf]

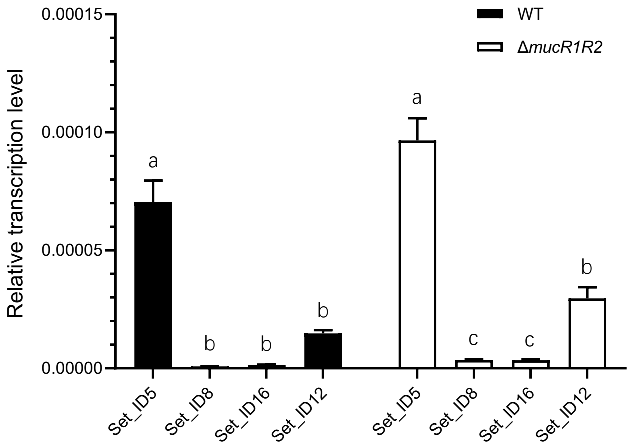

Fig. S6. RT-qPCR analysis of transposase mRNA levels in the wild-type strain and the Δ*mucR1R2* mutant. Different letters indicate significant difference in WT or the Δ*mucR1R2* mutant (ANOVA followed by Duncan's test; error bars represent SEM based on three biological replicates).
