# Supplementary material for: Intracellular common gardens reveal niche differentiation in transposable element community during bacterial adaptive evolution
Source: ISME J. 2022 Nov 24;17(2):297–308. doi: 10.1038/s41396-022-01344-2 (PMC9860058; doi:10.1038/s41396-022-01344-2)
Supplement: Supplementary file 7 — Figure S7 [file 41396_2022_1344_MOESM7_ESM.pdf]

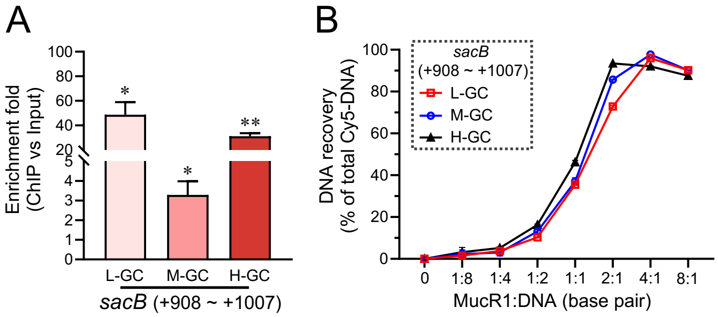

Fig. S7. ChIP-qPCR and Microscale thermophoresis analyses of the interaction between MucR1 and *sacB* of different GC content. (**A**) ChIP-qPCR showing that MucR1 binds the same *sacB* region (+908 ~ +1007) of different GC content *in vivo* in the pA replicon. Significant difference based on one-sample t-test is indicated. (**B**) Microscale thermophoresis (MST) showing that MucR1 can form DNA-MucR1-DNA bridging complex in a *sacB* region (+908 ~ +1007) of different GC% *in vitro* (Average ± SEM based on four technical replicates).
