# Supplementary material for: Intracellular common gardens reveal niche differentiation in transposable element community during bacterial adaptive evolution
Source: ISME J. 2022 Nov 24;17(2):297–308. doi: 10.1038/s41396-022-01344-2 (PMC9860058; doi:10.1038/s41396-022-01344-2)
Supplement: Supplementary file 8 — Figure S8 [file 41396_2022_1344_MOESM8_ESM.pdf]

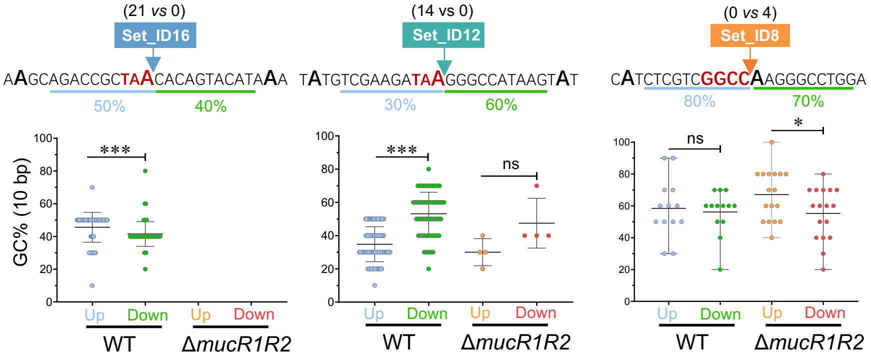

Fig. S8. GC content of 10 bp up- and down-stream sequences flanking the insertion sites of Set_ID16, Set_ID12, and Set_ID8. An insertion hotspot of Set_ID16 (between -30 and -29 nt of starting codon) and Set_ID12 (between +768 and +769 of M-GC *sacB*) in the wild-type SF2 derivatives, and that of Set_ID8 (between +237 and +238 of M-GC *sacB*) in the Δ*mucR1R2* background are shown. ns, not significant; *, $p < 0.05$; ***, $p < 0.001$; based on two-sample t-test.
