# Supplementary material for: Intracellular common gardens reveal niche differentiation in transposable element community during bacterial adaptive evolution
Source: ISME J. 2022 Nov 24;17(2):297–308. doi: 10.1038/s41396-022-01344-2 (PMC9860058; doi:10.1038/s41396-022-01344-2)
Supplement: Supplementary file 9 — Figure S9 [file 41396_2022_1344_MOESM9_ESM.pdf]

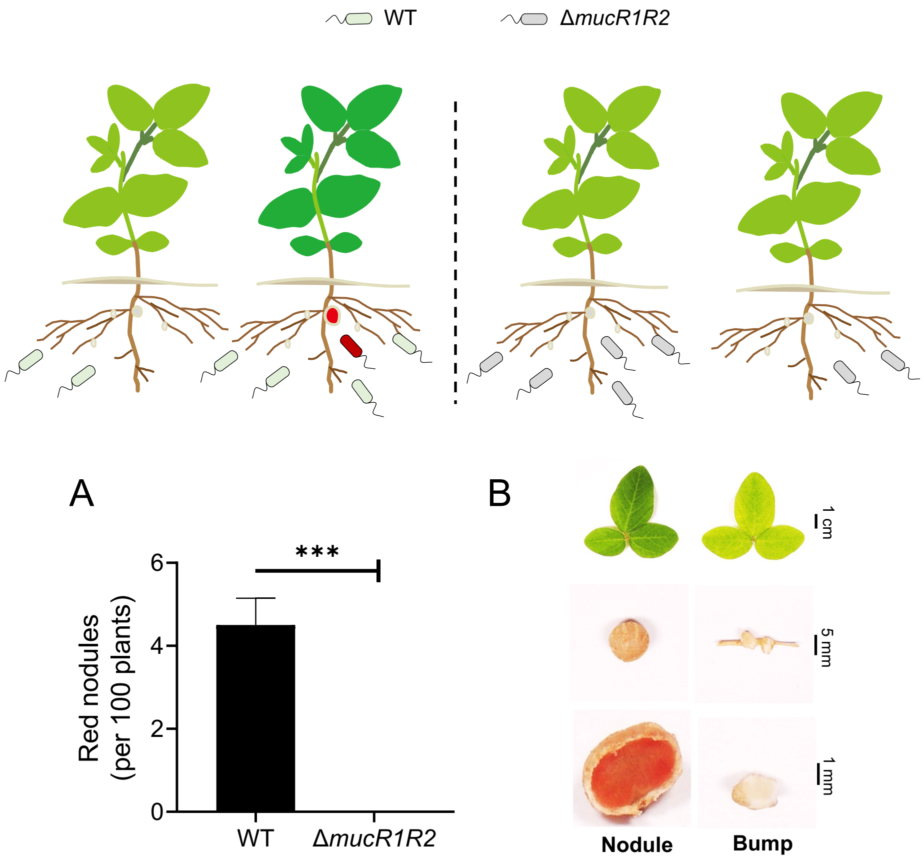

Fig. S9. Adaptive evolution of symbiotic compatibility of SF2 was impaired in the Δ*mucR1R2* mutant. (**A**) Average red nodule numbers per 100 soybean plants (*Glycine max* cv. JD17; 30 days post inoculation). ***, $p < 0.001$ (t-test; error bars represent SEM based on three independent experiments). The wild-type SF2 can evolve into compatible microsymbionts of *G. max* cv. JD17 mainly via IS insertion mutations in the gene cluster encoding type 3 secretion system and its effector protein (Zhao et al. 2018 ISME J). (**B**) The canonical red nodules formed by the compatible clones evolved from the wild-type SF2 were not found in the treatment of the Δ*mucR1R2* mutant.
